# Beam Steering Using Momentum-Reconfigurable Goubau Meta-Line Radiators

**DOI:** 10.1038/s41598-018-29507-2

**Published:** 2018-08-07

**Authors:** Xiao-Lan Tang, Qingfeng Zhang, Sanming Hu, Shangkun Ge, Yifan Chen, Hao Yu

**Affiliations:** 1 The Department of Electrical and Electronic Engineering, Southern University of Science and Technology, Shenzhen, 518055 China; 2 State Key Laboratory of Millimeter Waves, Southeast University, Nanjing, 210096 China; 3 The Department of Electronic and Computer Engineering, Hong Kong University of Science and Technology, Hong Kong, China; 4 The Department of Computer Science, University of Waikato, Hamilton, 3240 New Zealand

## Abstract

Spoof/designer surface plasmon polaritons (SPP) and Goubau line belong to the same category of single-conductor surface waveguide. They feature easy integration and high field confinement capability, and hence are good candidates for wave guiding and radiating at terahertz frequencies. Here, we propose a momentum-reconfigurable Goubau meta-line radiator that is capable of digitally steering its beam at a fixed frequency, in contrast to conventional SPP or Goubau line radiators relying on changing frequencies to steer beams. By periodically loading switchable meta-lines with *ON* and *OFF* states along the Goubau line, the modulation period and hence the momentum of Goubau line radiators can be dynamically controlled. The proposed Goubau line radiator is able to steer the main beam at a given frequency by independently switching *ON* or *OFF* each unit cell. As a proof of concept, we use line connection and disconnection to mimic *ON* and *OFF* state of the switch, respectively. Several radiators, representing different switching coding combinations, are fabricated and experimentally validated. Although this momentum-reconfigurable Goubau meta-line radiator is demonstrated at microwave frequency, it can be easily extended to terahertz frequencies.

## Introduction

Single-wire transmission lines (TLs), such as Goubau line^1,2^ and spoof/designer surface plasmon polaritons^3–9^, featuring groundless configurations, have attracted considerable attentions in the past few years owing to their ability of high electric field confinement and much lower transmission attenuation. In microstrip line and coplanar waveguide (CPW), electrical fields are largely confined in the dielectric substrate, leading to both dielectric and ohmic losses in the dielectric and ground planes. However, in Goubau lines, the electromagnetic fields highly confined to the metallic surface result in the propagation of a surface wave. The first single-wire TL was introduced by Sommerfeld^10^, demonstrating a non-radiating wave propagation in a cylindrical metallic conductor. Later, the Goubau line composed of a single conductor with a coated dielectric layer was proposed. Many research works have been carried out to demonstrate and validate strong electric field confinement and low-loss transmission properties of Goubau line in different frequency bands^11–13^. Since then, associated with their simple geometry, Goubau transmission lines were widely explored in other complex functionalities such as Goubau line radiators^14–16^.

Benefiting from their low-cost, light weight and simple fabrication features, the periodic radiators have found applications for large phased-array beam scanning used in microwave radar and communication systems. In some applications operating in a relatively narrow or predefined frequency band, steering beam directions without changing the frequencies is preferred. Many efforts have been made and several methods have been developed for beam scanning at a fixed frequency^17–29^. The technique of loading lumped capacitors was employed in a half-width^17^ and half-width leaky-wave radiators^18^, however the scanning angles were limited due to the capacitor values. In ref.^19^, the microstrip radiator was loaded by a number of stubs at both edges of microstrip. Another method is to use the PIN diodes to electronically control the beam^20,21^, but the resulting radiators allow only two discrete beam angles due to the two limited states of diodes. Nevertheless, no research works have been publicly reported to date on fixed-frequency beam steering of Goubau-line radiators.

In this work, we propose a momentum-reconfigurable Goubau meta-line radiator that is capable of digitally steering its beam at a fixed frequency. The radiation leakage is produced by loading period-reconfigurable meta-lines all through the Goubau line. By loading these meta-lines using *ON* or *OFF* state switches, we demonstrate theoretically and experimentally that a dynamic control over the momentum can be achieved. In this way, the main beam of the proposed radiator can steer continuously at a fixed frequency. This new concept of Goubau meta-line radiators using reconfigurable periodic modulations is experimentally demonstrated at microwave frequencies.

## Results

### Theoretical analysis and proof of the concept

**Figure 1:**
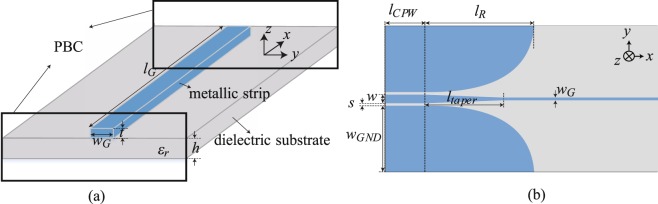
(**a**) 3D schematic view of a planar Goubau line. (**b**) Structure description of the CPW-to-Goubau-line tapered transition.

Planar Goubau lines, formed by a single rectangular-shaped conductor lying on a flat dielectric slab, are particularly promising because of their simple geometry adapted for complex integrated schemes with high functionalities. The schematic of Goubau line is shown in Fig. 1(a), where the parameters *w*_*G*_, *l*_*G*_, *t* and *h* denote the line width, the line length, the strip thickness and the substrate thickness, respectively. In the practical implementation, an impedance and mode conversion structure is required to allow the connection to the conventional two-conductor transmission lines. For this purpose, the ground planes are gradually tapered on both sides to convert smoothly the modes supported in 50 Ω transmission line and Goubau line. This transition structure allows also 50 Ω impedance matching. An example of CPW-to-Goubau-line converter is shown in Fig. 1(b), where the parameters *w*, *s*, *w*_*GND*_, *l*_*CPW*_, *l*_*taper*_ and *l*_*R*_ denote CPW line width, CPW line gap, CPW ground width, the length of CPW ground plane, of the taper and of the exponentially-grading CPW ground plane, respectively.

**Figure 2:**
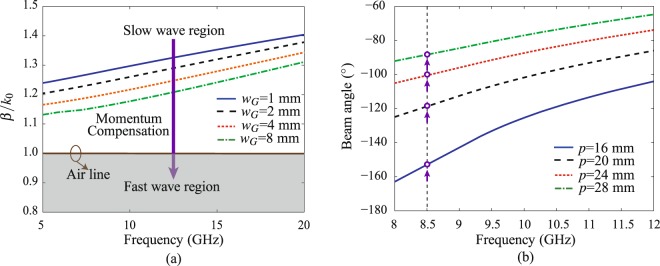
(**a**) Dispersion curve of the planar Goubau line of different line width *w*_*G*_(*l*_*G*_ = 5 mm, *h* = 1.52 mm and *t* = 1.8 um) on a Rogers 4003C substrate (*ε*_*r*_ = 3.38, tan *δ* = 0.0027). (**b**) The calculated beam scanning angles of the periodically modulated Goubau line radiators with different modulation periodicity *p*.

To illustrate the propagation modes, the dispersion curve of the fundamental mode of Goubau line of different *w*_*G*_ (*l*_*G*_ = 5 mm, *h* = 1 mm and *t* = 18 um) on a Rogers 4003 C substrate (*ε*_*r*_ = 3.38, tan *δ* = 0.0027) is plotted in Fig. 2(a). It is calculated by enforcing periodic boundary conditions (PBC) on both ends of the Goubau line, as shown in Fig. 2(a). It shows that the slow-wave (*β*/*k*_0_ > 1) propagation mode is dominant in Goubau line, which is larger than that of the air (*β*/*k*_0_ = 1). When the Goubau line width becomes narrower, the wave gets slower and hence achieves a stronger field confinement around the surface. The fundamental mode of Goubau line is bounded (i.e. a non-radiating slow-wave mode). It does not radiate because of the momentum mismatch with the wave in the air. To excite its radiating modes, one way is to introduce deliberately periodic modulations in the guiding structure. These periodic modulations can create an infinite number of space harmonics characterized by its wavenumber *β*_*n*_ (*n* = 0, ±1, ±2, …), in which some space harmonics can be fast under specified conditions. A typical radiation leaky mode is the space harmonic *n* = −1. Therefore, for a leaky-wave radiator, the main direction of the radiated beam *θ* is approximately computed by

1 $$\sin (\theta )={\beta }_{-1}/{k}_{0},$$

where *k*_0_ denotes the wavenumber in the free space. Note that, the momentum (in proportion to wavenumber) ratio of the space harmonic and free space determines the beam angle.

For a periodically modulated Goubau line, the first space harmonic is *β*_−1_ = *β*_0_ − 2*π*/*p*, with *p* the modulation period and *β*_0_ denotes the wavenumber of the unmodulated Goubau line. After applying *k*_0_ = 2*πf*/*c*_0_ to Eq. (1), where *c*_0_ is the light speed in the air and *f* is the operation frequency, one obtains

2 $${\theta }_{f,p}=\arcsin (\frac{{\beta }_{0}}{{k}_{0}}-\frac{{c}_{0}}{fp}),$$

The direction of the main beam in the periodic radiators depends on both the periodicity of the added modulations *p* and the excitation frequency *f*, as shown in Eq. (2). Note that when the modulation period is fixed, the beam angles steer with the frequency, leading therefore to a frequency-steerable radiation pattern. In contrast, once the operation frequency is determined, the modulation period becomes the key parameter to determine the beam direction of radiators. Therefore, the fixed-frequency beam-steering performance may be achieved by dynamically changing the periodicity of the modulated Goubau-line.

Figure 2(b) plots calculated beam scanning properties of Goubau-line-based (*w*_*G*_ = 1 mm) radiator using Eq. (2). Firstly, it is shown that the frequency-scanning property can be obtained in a modulated Goubau line radiator with a fixed period. Most importantly, changing the modulation periods allows main beam scanning at a fixed frequency and also allows the main beam to be fixed whereas the frequency is changed. For example, the beam angle scans from −159.0° to −88.2° with a wide scanning range of +70.8° at a frequency of 8.5 GHz when the modulation period varies from 16 mm to 28 mm.

**Figure 3:**
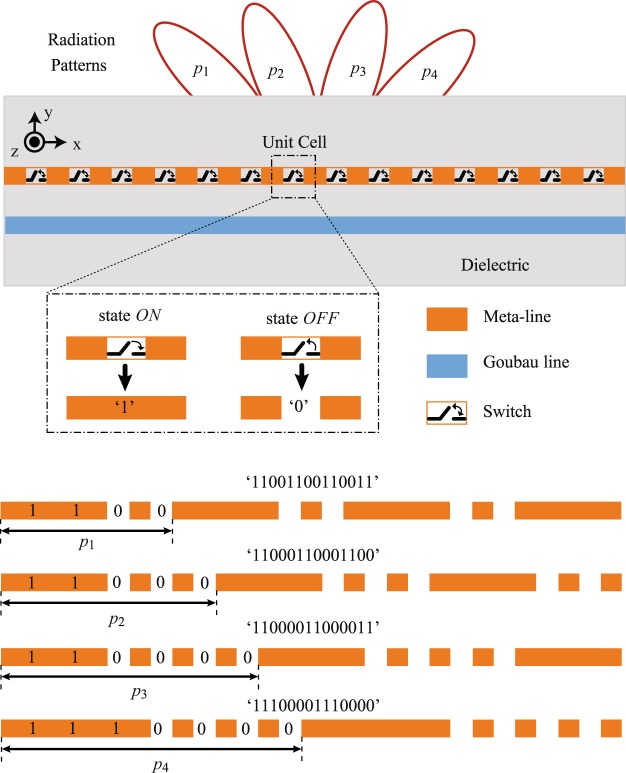
Schematic description of a modulated periodic radiator based on Goubau line with switchable meta-lines for beam steering.

To realize a dynamic variation of the modulation period in Goubau line radiators, we propose an electronic approach using the switches, as illustrated in Fig. 3 (mode conversion structures is not shown for simplicity). The Goubau line profile is modulated by the meta-lines placed at a certain distance away, but interconnected by the sequent switches. These switches can be connected or disconnected in an independent manner, which corresponds to an electronic state of ‘1’ (i.e. switch *ON*) or ‘0’ (i.e. switch *OFF*), respectively. In an approximate way, when the switch is turned *ON*, the length of the meta-line is “electronically” extended whereas the *OFF* state is achieved by leaving a gap between these lines.

One unit cell of radiator consists of a meta-line and a switch where the state of switches in each period can be switched between ‘1’ and ‘0’. Each modulation consists of *m* unit cells (*m* = 1, 2, 3, …) which determines the period of the modulation *p*_*m*_. The radiation pattern of the steerable radiator depends on *p*_*m*_ and the switch states in each period. It should be noticed that *m* unit cells in one period result in 2^*m*^ different switching coding combinations. For instance, an example of 4 unit cells (i.e. modulation period *p*_1_) with a coding “1100” is investigated in Fig. 3. By choosing different switching combinations, the main beam of the radiator may be steered even at a fixed frequency.

**Figure 4:**
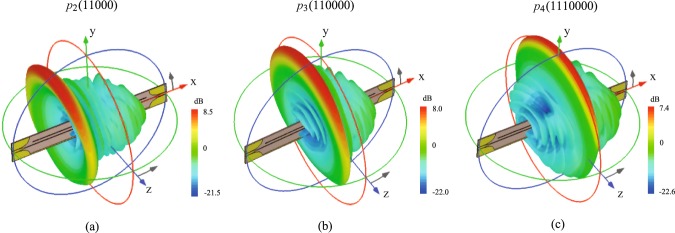
The simulated 3D radiation patterns of the modulated radiators of different unit cells at 8.5 GHz. (**a**) *p*_2_ in switch coding “11000”. (**b**) *p*_3_ in switch coding “110000”. (**c**) *p*_4_ in switch coding “1110000”.

To better show the scanning property of these Goubau line radiators of different modulation periods at a fixed frequency, we compare the 3D radiation patterns at the same frequency of 8.5 GHz in the same scale, as shown in Fig. 4. It can be clearly seen that the scanning angles change with the modulation period keeping a flat radiation gain whereas the frequency is fixed.

### Design validation, simulations and experimental results

**Figure 5:**
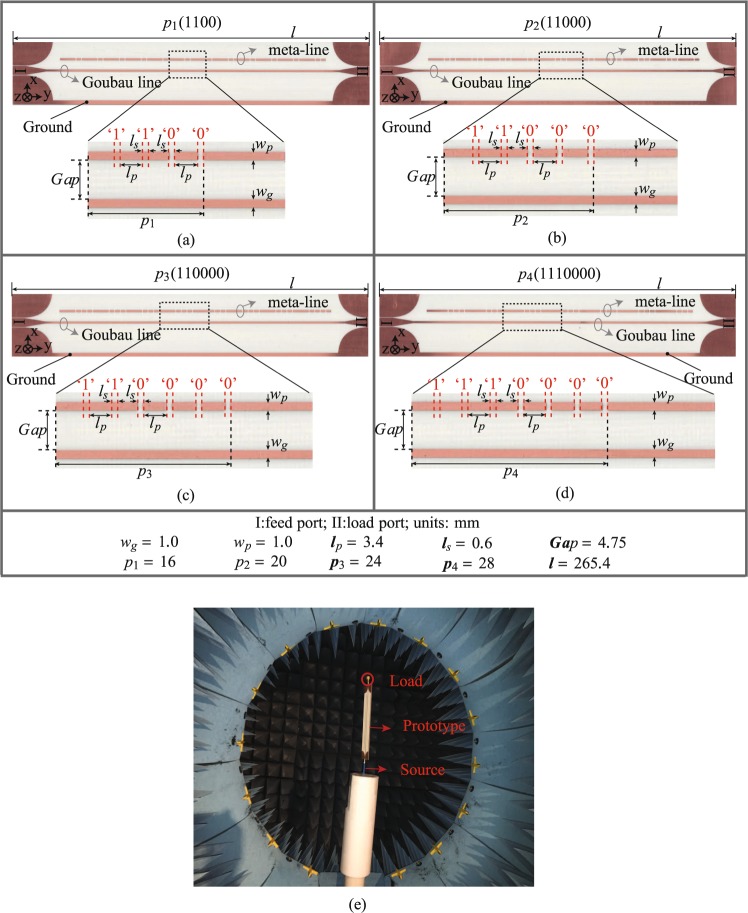
Fabricated prototypes of reconfigurable radiators with their physical dimensions. (**a**) *p*_1_ in switch coding “1100”. (**b**) *p*_2_ in switch coding “11000”. (**c**) *p*_3_ in switch coding “110000”. (**d**) *p*_4_ in switch coding “1110000”. (**e**) The photograph of experimental setup for radiation pattern measurement.

As a proof of concept, a periodically-modulated Goubau line radiator composed of 48 unit cells (i.e. 48 meta-lines and switches) are used in simulation for optimization to establish the feasibility of the fixed-frequency beam scanning performance. To experimentally validate the proposed radiator, we fabricate four modulated radiators of different modulation periods, as shown in Fig. 5. The total length of radiators is 265.4 mm and the corresponding modulation periods are 16 mm, 20 mm, 24 mm and 28 mm, respectively. For simplicity of comparison, one specific switch states combination is considered for each radiator, in which a digital coding of“1100”, “11000”, “110000” and “1110000” is chosen for *p*_1_, *p*_2_, *p*_3_ and *p*_4_, respectively. These switch coding combinations are also shown in Fig. 3. All the prototypes are implemented on 1.52-mm-thick Rogers 4003C substrates (*ε*_*r*_ = 3.38 ± 0.05, tan *δ* = 0.0027). The optimized physical dimensions of CPW-to-Goubau-line transition are: *w* = 4.5 mm, *s* = 0.3 mm, *w*_*GND*_ = 15 mm, *l*_*CPW*_ = 10 mm, *l*_*taper*_ = 15 mm and *l*_*R*_ = 20 mm and the other physical dimensions are listed in Fig. 5. Note also that, the bottom ground planes of all radiators are deliberately connected to improve the radiation performance. Figure 5(e) shows the experimental setup for radiation pattern measurement of all the prototypes.

**Figure 6:**
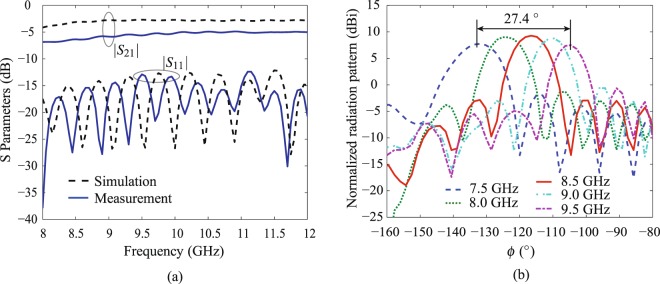
Performance of the modulated radiators for period *p*_2_ in switch coding “11000”. (**a**) The simulated and measured scattering parameters. (**b**) The measured far-field E-plane radiation patterns.

**Figure 7:**
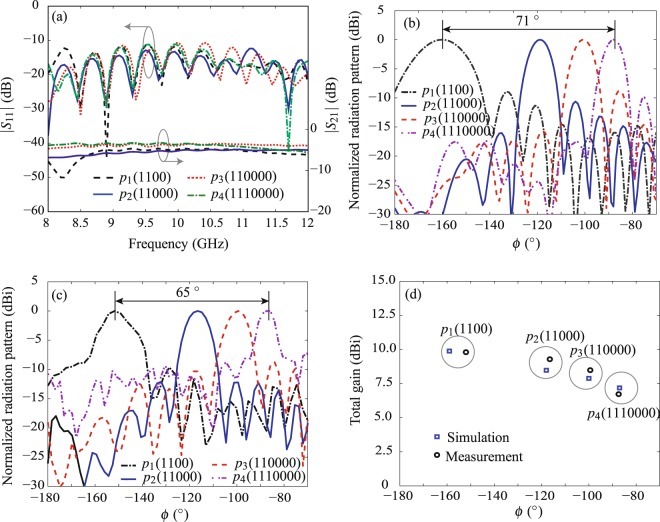
Performance comparison of the modulated radiators of different modulation periods in Fig. 5 at 8.5 GHz. (**a**) The measured scattering parameters. (**b**) The simulated far-field E-plane radiation patterns. (**c**) The normalized E-plane radiation patterns in measurement. (**d**) The measured and simulated gains and beam angles.

The S-parameters of the implemented radiators were measured using an Agilent network analyzer PNA E5071C. Figure 6(a) shows the comparison of simulated and measured scattering parameters of the fabricated radiator *p*_2_ (11000) and Fig. 7(a) shows the measured S-parameters for all fabricated radiators. The measured results agree well with the simulated ones. A slight frequency shift is observed which is probably due to the tolerance in dielectric permittivity (3.38 ± 0.05). As shown in Fig. 7(a), the measured transmission coefficients *S*_21_ for all structures are below −10 dB within 8–12 GHz. Note that, the measured reflection coefficients *S*_11_ for all radiators is lower than −12 dB, showing that a good impedance and momentum matching is achieved.

A commercial measurement system Satimo Starlab was employed to measure the near-field performance of radiators, which were then numerically computed to the far-field radiation patterns. The measured frequency-dependent E-plane radiation patterns of the fabricated radiator *p*_2_ (11000) is shown in Fig. 6(b). The frequency scanning performance is clearly shown with a scanning angle of 27.4° within 7.5–9.5 GHz. In contrast, Fig. 7(b,c) show respectively the normalized simulated and measured far-field E-plane radiation patterns at a fixed frequency for radiators of different modulation periods illustrated in Fig. 5. At 8.5 GHz, these radiators are steerable from −154.4° to −87.4° with a scanning range of 65° in the measurement. Note that there is a beam shift compared with the theoretical prediction using Eq. (2) (i.e. calculated value 70.8°) and also with the simulation results (i.e. 71° shown in Fig. 7(b)). This measured beams angle shift is also observed in Fig. 7(d), which shows the performance comparison of measured and simulated realized gains and beam angles of fabricated radiators. The difference is probably due to the measurement setup errors.

## Discussion

Four periodic Goubau meta-line radiators using switched modulations are designed and compared. These radiators exhibit frequency scanning properties and also provide the possibility of beam scanning at a given frequency. It has been theoretically and experimentally shown that the modulation periods can be dynamically varied by using the *ON* or *OFF* state of the switches, which enables a flexible control of the main beam direction of radiators. Note that the measurement results agree well with the simulations, demonstrating a good fixed-frequency beam steering ability. These radiators can find its applications for large phased-array systems such as microwave radar and communication systems of narrow band. In addition, by further employing the PIN diodes as the switch function in the structure, truly reconfigurable Goubau-line-based radiators can be obtained in fabrication. However, it should be noticed that adding the PIN diodes and its corresponding biasing circuits introduces parasitic parameters (i.e. parasitic resistance, inductance and capacitance). Simulations show that the performance of radiators in terms of realized gain could be deteriorated. Taking advantage of the low-loss transmission of Goubau line as the frequency increases, high-performance reconfigurable radiators working in THz band may be expected with the scaling-down of physical dimensions. Moreover, the development of advanced nanotechnologies enables the application of medical imaging systems where high-performance integrated THz radiators are required.

## Methods

To perform the electromagnetic simulations of all the proposed radiators, a commercial software CST Microwave Studio is employed. The dispersion curves of the Goubau line are numerically computed using the Eigenmode Solver by enforcing a periodic boundary condition on both sides of the line. The S-Parameters results of the radiators are simulated by a time-domain solver of CST tool and measured using a network analyzer (Agilent PNA E5071C). To obtain the near-filed performance and far-field radiation patterns of these radiators in microwave bands, a commercial measurement system Satimo Starlab is employed.
